# Evolution of parasitoid host preference and performance in response to an invasive host acting as evolutionary trap

**DOI:** 10.1002/ece3.9030

**Published:** 2022-07-04

**Authors:** Astrid Kruitwagen, Leo W. Beukeboom, Bregje Wertheim, G. Sander van Doorn

**Affiliations:** ^1^ Groningen Institute for Evolutionary Life Sciences University of Groningen Groningen The Netherlands

**Keywords:** biological control, ecological trap, evolution, exotic species, host–parasitoid interactions, parasitism

## Abstract

The invasion of a novel host species can create a mismatch in host choice and offspring survival (performance) when native parasitoids attempt to exploit the invasive host without being able to circumvent its resistance mechanisms. Invasive hosts can therefore act as evolutionary trap reducing parasitoids' fitness and this may eventually lead to their extinction. Yet, escape from the trap can occur when parasitoids evolve behavioral avoidance or a physiological strategy compatible with the trap host, resulting in either host‐range expansion or a complete host‐shift. We developed an individual based model to investigate which conditions promote parasitoids to evolve behavioral preference that matches their performance, including host‐trap avoidance, and which conditions lead to adaptations to the unsuitable hosts. The model was inspired by solitary endo‐parasitoids attacking larval host stages. One important aspect of these conditions was reduced host survival during incompatible interaction, where a failed parasitization attempt by a parasitoid resulted not only in death of her offspring but also in host killing. This non‐reproductive host mortality had a strong influence on the likelihood of establishment of novel host–parasitoid relationship, in some cases constraining adaptation to the trap host species. Moreover, our model revealed that host‐search efficiency and genetic variation in host‐preference play a key role in the likelihood that parasitoids will include the suboptimal host in their host range, or will evolve behavioral avoidance resulting in specialization and host‐range conservation, respectively. Hence, invasive species might change the evolutionary trajectory of native parasitoid species, which is important for predicting biocontrol ability of native parasitoids towards novel hosts.

## Graphical abstract

Invasive species can act as an evolutionary trap for parasitoids when they are unsuitable for reproduction but indistinguishable from suitable hosts. Such incompatible interactions reduce parasitoids’ fitness and can reduce the viability of the population. Using an individual based model, we show how genetic variation in host preference and performance influence the evolution of generalization and specialization in response to an evolutionary trap.

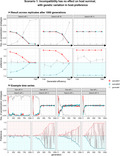

## 1 INTRODUCTION

Parasitoids are insects that lay eggs in or on other insects, and whose immature stages develop in or on a host that is eventually killed. They however sometimes accept hosts for oviposition that are unsuitable for their offspring to survive (Heimpel et al., 2003; Thompson, 1988). One explanation of such “bad motherhood” is the invasion of novel host species for which native parasitoid species do not have pre‐adapted mechanisms to overcome host resistance, and/or to recognize these hosts as unsuitable (Thompson, 1988; Yoon & Read, 2016). For instance, native parasitoids are reported to attack the exotic species, *Harmonia axyridis, Halyomorpha halys, Drosophila suzukii*, and *Cydalima perspectalis* but their offspring perform relatively poorly with low survival (Konopka et al., 2018; Martini et al., 2019; Romero et al., 2020). Exploiting suboptimal hosts can therefore result in reduced fitness when parasitoids lose resources, time and/or eggs, exploring and attacking them. When parasitoids exploit suboptimal hosts even when alternative suitable ones are present, the host is considered an “evolutionary trap” (Schlaepfer et al., 2002; Schlenke et al., 2007). Evolutionary traps can have large ecological impact on ecosystems: they can reduce the parasitoid population size and ultimately drive it to local extinction (Kokko & Sutherland, 2001; Yoon & Read, 2016).

Maladapted parasitoid populations can be “rescued” by undergoing evolutionary change. Studies have shown that some parasitoid populations harbor genetic variation for host‐choice and host use/virulence (Benoist et al., 2020; Desjardins et al., 2010; Henter, 1995; König et al., 2015). This would allow parasitoids to escape from an evolutionary trap by evolving avoidance behavior or physiological compatibility (i.e., improved host use/performance) (Keeler & Chew, 2008). Both mechanisms would promote parasitoid persistence, but only adaption to efficient utilization of the novel host would reduce the impact of the invader and would thus be favored from a conservation and biological control perspective. Note that alterations in host‐choice and host‐use might also arise due to plastic changes; parasitoids might avoid suboptimal host (patches) through learning or overcome host resistance through superparasitism. Insight in the response to an evolutionary trap is also relevant for the evolution of host specialization or host‐range expansion. Inclusion of the suboptimal host into the parasitoid's repertoire of hosts species can eventually result in specialization and host‐shift to this novel host, whereas behavioral avoidance results in physiological host‐range conservation.

Models that focus on host–parasitoid dynamics typically assume that a compatible host–parasitoid interaction results in host mortality and yields parasitoid offspring, whereas an incompatible interaction (such as a host trap) results in host survival and no parasitoid offspring (Hassell, 2000; Hassell & Pacala, 1990; Heimpel et al., 2003). However, a mismatch in parasitization strategy and host suitability (incompatibility) can also result in “non‐reproductive host mortality,” that is, parasitoids kill the unsuitable host but their offspring do not develop (Abram et al., 2016). This has, for example, been documented in native scelionine egg parasitoid wasps (Hymenoptera: Scelionidae) attacking the invasive agricultural pest *Halyomorpha halys* (Abram et al., 2014, 2016) and figitid larval parasitoids (Hymenoptera: Figitidaea) attacking *Drosophila suzukii* (Kruitwagen et al., 2021). Non‐reproductive host killing is thus a third outcome of a host–parasitoid interaction, but has received little attention in host–parasitoid studies despite its common nature (Abram et al., 2019).

Empirical and theoretical studies show that non‐reproductive host killing can influence the population size of the unsuitable host when suitable hosts are present to sustain the parasitoid population (Abram et al., 2016; Huang et al., 2017; Kaser et al., 2018; Münster‐Swendsen, 2002). However, the inclusion of the unsuitable host in the parasitoid's repertoire can reduce the overall reproductive success of the parasitoid and can consequently feedback on host–parasitoid population dynamics and relative host species abundance via complex direct and indirect interactions (Kaser et al., 2018). For instance, time and/or eggs lost by attacking unsuitable hosts can reduce parasitoid population size and release the suitable hosts from parasitism (“enemy release”). Such release could in return also be positive: for example, reduced parasitism by scelionine egg parasitoids when attacking the unsuitable host *H. halys* could facilitate population growth of its suitable stink bug host *Podisus maculiventris*, which is an important biocontrol agent. As host availability and suitability have a direct impact on parasitoid reproductive success, they can also shape the evolution of the parasitoid behavior and parasitization strategies. Hence, attacking and killing unsuitable hosts can change host population abundance of both suitable and unsuitable hosts (Heimpel et al., 2003; Kaser et al., 2018) and might thus alter the strength and direction of selection in response to the host trap.

The impact of variation in host‐suitability on parasitoid ecology and evolution has previously been modeled in 1‐host/1‐parasitoid (Fellowes & Travis, 2000; Sasaki & Godfray, 1999; Tuda & Bonsall, 1999) and 2‐host/1‐parasitoid systems (Heimpel et al., 2003; Kaser et al., 2018; Tuda & Bonsall, 1999). However, it is unknown how parasitoid induced non‐reproductive host mortality interacts with genetic variation in host preference behavior and parasitization strategy, that is, their physiological compatibility with different host species. Using an individual‐based model, we here address the questions: (1) how does genetic variation for host preference and parasitization strategy influence the evolution of generalization and specialization in response to an evolutionary trap and (2) how does non‐reproductive host mortality (i.e., an incompatible interaction that results in host death without parasitoid offspring) influences the evolutionary response of the parasitoid? We explore the evolution of parasitization strategy and preference behavior in a 2‐host/1‐parasitoid system under various costs of exerting host preference and generalist parasitization efficiencies, to identify conditions that would favor trap avoidance and/or adaptation to the unsuitable host. The model was inspired by solitary koinobiont endo‐parasitoids attacking larval host stages, such as the larval parasitoid *Leptopilina heterotoma*.

## 2 MATERIALS AND METHODS

### 2.1 Model description

We simulate the evolution of two evolving traits, host preference behavior and parasitization strategy (i.e., physiological compatibility with two host species), in a population of parasitoids that has access to an original host species (host species 1) and a novel invader (host species 2). Generations are discrete and non‐overlapping for both host species and the parasitoid; we denote their population densities at the start of generation *t* by *H*_1_(*t*), *H*_2_(*t*), and *P*(*t*), respectively.

### 2.2 Host population dynamics

The environment is structured into patches that each contain a small subpopulation of host individuals. We assume that the two host species occur in the same area but in two different microhabitats, so that they occur in separate patches and do not compete for resources with each other. The density of each host population is regulated at the global scale by density‐dependent survival, which acts before the start of each parasitoid generation. The surviving hosts reproduce, and distribute their offspring randomly over the host patches for their species, after which the host adults die. The intrinsic population dynamics of the host are described by a stochastic variant of the Ricker model (Ricker, 1954), where the total number of offspring in a local subpopulation of host species *i* (*i* = 1 or 2) is distributed following a Poisson distribution with mean:

$$\lambda_{i}\left(t\right)=\frac{1}{n_{i}}r_{i}\;H_{i}\left(t\right)\;e^{-\frac{H_{i}\left(t\right)}{K_{i}}}.$$

Here, *n*_*i*_ is the number of patches for host species *i* in the environment, *r*_*i*_ is the maximum per‐capita offspring production rate, $H_{i}\left(t\right)$ denotes the total number of adult host individuals before density regulation (which is equal to the number of surviving hosts at the end of the previous time step), and $K_{i}$ reflects the carrying capacity of host species *i* with respect to within‐species competition (Table 1); this parameter determines the host population density at equilibrium in the absence of parasitization, $H_{i}^{*}=K_{i}∙\ln\left(r_{i}\right)$.

**TABLE 1:** Parameters and initial values used in simulations

Parameter	Interpretation	Parameter value(s)
*Parasitoid*		
$\mu_{q}$	Mutation rate of host‐preference	0;0.001
$\mu_{S}$	Mutation rate of parasitization strategy	0;0.01
$\tau_{d}^{P_{1}};\tau_{d}^{P_{2}}$	Development time of parasitoid offspring for specialists (S = 1, S = 2)	20
$\tau_{d}^{P_{3}}$	Development time of parasitoid offspring for generalists (S = 3)	46.51; 45.45; 44.44; 43.48; 42.55
$\tau_{s}$	Search time, time for parasitoids to find a patch with hosts	3;7;11
$\tau_{a}$	Time after which the parasitoid abandons the patch when failing to locate another host	1
$\tau_{e}$	Host–parasitoid encounter rate	0.02
$\tau_{r}$	Time needed for parasitoid to recover after foraging and localizing the next host‐patch	0.5
*P(t)*	Initial parasitoid population size	500
*Host*		
*r*_*1*_	Growth rate host 1	4
*r*_*2*_	Growth rate host 2	4
*K*_1_	Carrying capacity coefficient host 1	1000
*K*_2_	Carrying capacity coefficient host 2	1000
$\tau_{d}^{H_{i}}$	Time for a host to mature	10
*n*_*1*_	Number patches of host 1	20
*n*_*2*_	Number patches of host 2	20

The developing host offspring that have been deposited in a patch can be parasitized during a vulnerable period in their development. The host survives if no parasitoid successfully parasitizes the host before the end of the sensitive period. For simplicity, we model host development as a first‐order process, so that the length of the sensitive period can be sampled from an exponential distribution with mean $\tau_{d}^{H_{i}}$, the average length of the sensitive developmental period for host *i*. Here, and also later on, more sophisticated waiting time distributions can be used if desirable, for example, based on available data, but at the cost of introducing additional parameters. However, irrespective of how waiting times are modeled precisely, variation in the length of the sensitive period introduces variation in the vulnerability of hosts to parasitoid attack, which is an important factor promoting the stabilization and persistence of host–parasitoid systems (Hassell, 2000; Hassell & Pacala, 1990).

### 2.3 Parasitoid behavior

**FIGURE 1:**
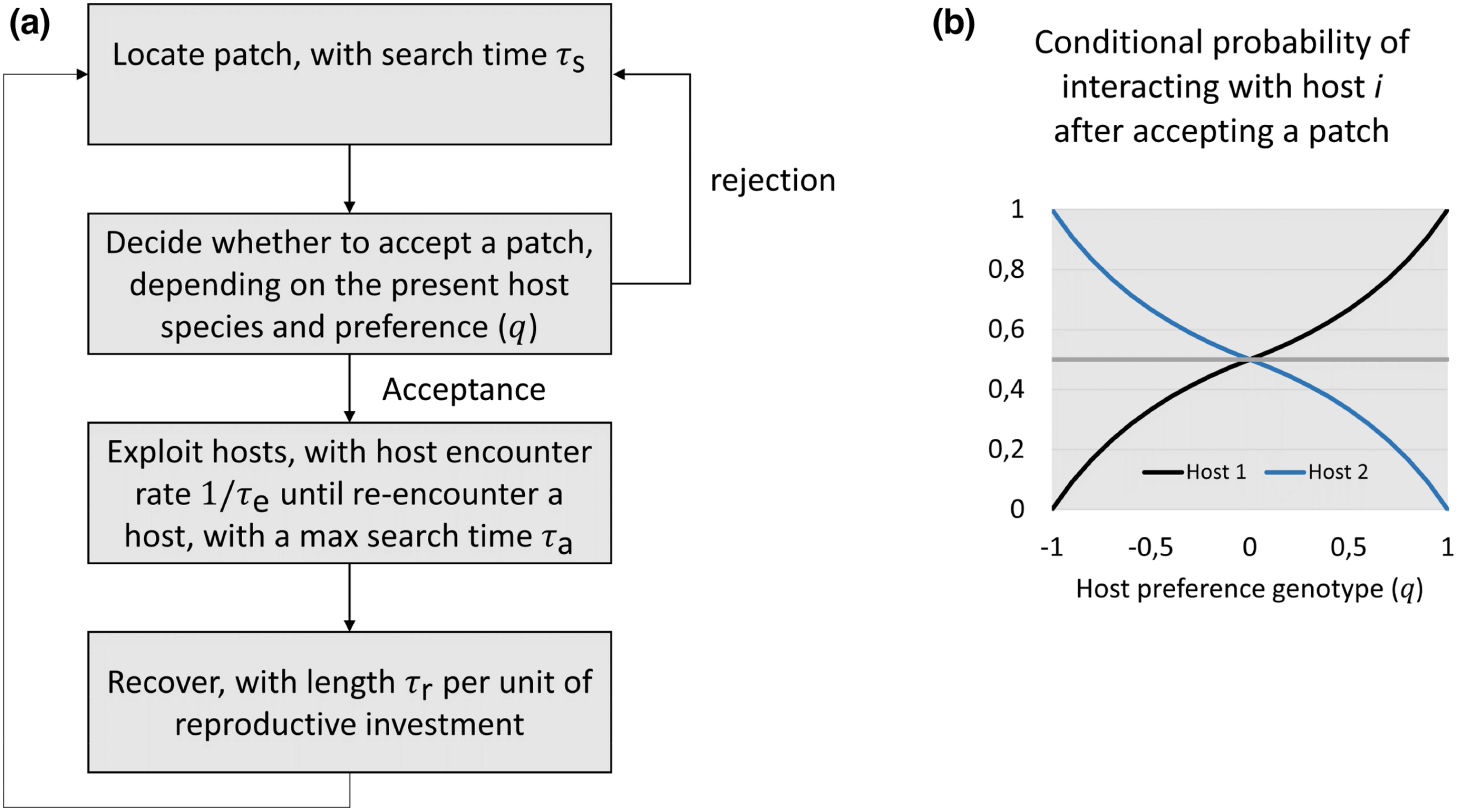
Stages of parasitoid host‐searching behavior (a) and the effect of parasitoid host preference genotype (*q*) on the probability of interacting with host species *i* after it has accepted a patch assuming equal numbers of host patches of each host species (b). When the probability equals 0.5, parasitoids interact with both host species with equal probability and thus select hosts at random (horizontal gray line). Hence, host preference q=0 indicates that parasitoids accept all host patches (i.e., random host searching), whereas, on the extremes of the scale, individuals with q=+1 or q=−1 exclusively accept patches occupied by host species 1 or 2, respectively, and thus avoid the other host

Parasitoid reproductive success is taken to be limited by the ability to locate and exploit host patches (i.e., parasitoids are limited by time, not primarily by the availability of resources needed for egg‐production). This is relevant as most parasitoids seem to live relatively short and die before exhaustion of all their eggs and/or are able to replenish their egg supply during their life (Ellers et al., 2000; Wajnberg, 2006; Wajnberg et al., 2016). In the individual‐based simulations, we therefore keep track of the time budget of each parasitoid individual as it progresses through consecutive stages of the parasitization cycle (Figure 1a).

The first step in the parasitization behavior is that the parasitoid must search the environment to locate a host patch. Searching parasitoids are assumed to move through the environment randomly and encounter host patches at a constant rate $1/\tau_{s}$, where $\tau_{s}$ represents the average searching time needed to locate a host patch. After locating a patch, the parasitoid may decide to reject it, depending on which host species is occupying the patch and the host preference trait of the parasitoid individual, q, a quantitative character that can range in value on a continuous scale between −1 and +1 (Figure 1, Table 1). In particular, the probability that the parasitoid rejects the patch is given by:

$$\mathrm{Pr}\left[\text{reject patch}\right]\left(q\right)=\left\{\begin{array}{cc} \max\left(0,-q\right) & \text{if patch is occupied}\;\mathrm{by}\;\text{host}\;1\\ \max\left(0,+q\right) & \text{if patch is occupied}\;\mathrm{by}\;\text{host}\;2 \end{array}\right.$$

Accordingly, individuals with q=0 accept all patches (i.e., they select a host patch at random), whereas, on the extremes of the scale, individuals with q=+1 or q=−1 exclusively accept patches occupied by host species 1 or 2, respectively, and thus avoid the other host (Figure 1b). Note that such selectivity comes at the costs of an increase in the expected search time needed to locate an acceptable patch (a parasitoid that rejects a patch has to resume searching).

Once an individual accepts a host patch, it proceeds to exploit the available hosts living there (Figure 1a). Parasitoids encounter hosts in a random sequence, and continue to search the patch until they encounter a host individual for the second time, or if they have been searching for longer than $\tau_{a}$ time units since their last encounter with a host individual ($\tau_{a}$ is the time threshold for abandoning the patch when failing to locate another host). Time intervals between encounters with a given host individual are drawn from an exponential distribution with mean $\tau_{e}$ (i.e., $1/\tau_{e}$ corresponds to the per‐capita host–parasitoid encounter rate). As a consequence of the stochasticity in the order and timing of host encounters, parasitoids tend to interact with a variable subset of the host individuals in a patch, rarely with all of them. Note, we thus assumed that hosts are distributed within the patch and are not clustered together. This can apply to parasitoids attacking the larval stage, but not egg masses since parasitoids do not require additional search time between encountering individual eggs once they have found the egg mass.

If a host is found by a parasitoid before it has completed the sensitive period of development ($\tau_{d}^{H_{i}}$), it will be parasitized, incrementing the parasitoid's reproductive investment by one unit. The parasitoid's reproductive investment reflects energy invested in parasitization and egg production. Hosts can be parasitized multiple times; we assume that parasitoids do not discriminate against hosts that were previously attacked by another individual. Hence, our model allows for superparasitism: a host can be attacked multiple times by different conspecific females. The incidence of self‐superparasitism will be low as we assumed parasitoids to leave a patch once it encounters a host individual for a second time. Many parasitoids are indeed reported to be able to discriminate between already parasitized hosts (e.g., Ueno & Tanaka, 1994; Varaldi et al., 2005). Conspecific superparasitism is relevant as theoretical and empirical studies show that this can occur in parasitoid wasps, including *L. heterotoma*, when the parasitization mark for example only last shortly and/or is not detectable by conspecific females (Hofsvang, 1988; Van Alphen & Visser, 1990; Visser et al., 1992), or even as an adaptive strategy for optimal patch usage (Visser et al., 1992).

After leaving a host patch, parasitoids need to recuperate for a period of time to restore their energy reserves allocated to reproduction: we assumed that this recovery period consists of a sum of exponentially distributed waiting times, each with expected length $\tau_{r}$ for each unit of reproductive investment that the individual spent in the previous host patch (Figure 1a, Table 1). After recovering from previous reproductive investment, parasitoids are allowed to resume their search for another host patch, until no exploitable hosts are available in the environment anymore. This thus corresponds to a Holling type 2 functional response, in which the attack rate per host individual decelerates with increasing host density due to an increase in the total host handling time.

### 2.4 Parasitoid offspring development

**FIGURE 2:**
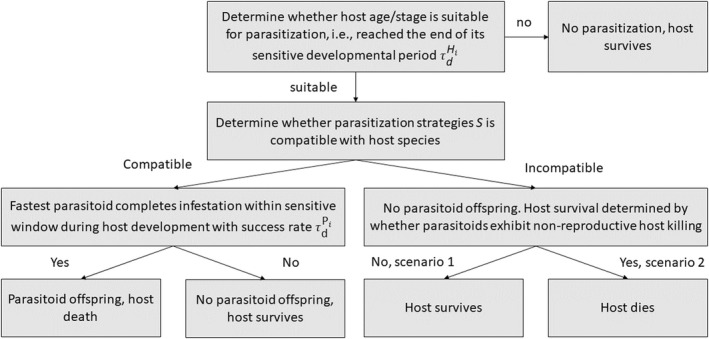
Stages of host exploitation by parasitoid wasps (parasitization) and the outcome of host–parasitoid interactions in terms of host and parasitoid offspring survival. When the parasitoid is incompatible with the host, two different scenarios are considered when the parasitoid exploits the host: Under scenario 1 the parasitoid offspring dies but the host survives, whereas under scenario 2 both the parasitoid offspring and host die

The outcome of the host–parasitoid interaction is dependent on the parasitization strategy (*S*) of the parasitoid, and the timing of host and parasitoid offspring development (Figure 2). In addition, we consider two different scenarios in modeling incompatible host–parasitoid interactions: one with non‐reproductive host‐killing, the other without (Table 2, Figure 2). The parasitization strategy *S* is modeled as a discrete character, with three possible trait values, reflecting the following tactics: parasitoids with S=1 or S=2 are specialist parasitoids that exhibit specific adaptations to, respectively, host species 1 or 2, and that are incompatible with the other host species; parasitoids with S=3 are generalists that are compatible with both hosts. As explained below, we assume that the broader host range of these generalists trades‐off against a lower rate of development efficiency of their offspring in either host species, resulting in a lower offspring survival probability (Straub et al., 2011).

**TABLE 2:** Overview of potential outcome of host–parasitoid interactions under two different scenarios. Parasitization strategy (*S*) 1 or 2, are specialist parasitoids only compatible with host 1 and 2 respectively, and *S* = 3 are generalist parasitoids compatible with both host species (host 1 and 2)

Parasitization strategy *S*	Host species	Outcome^a^
*Compatible interactions*		
1	1	Fast parasitoid development^b^; emergence of parasitoid offspring; host dies.
2	2	
3	1, 2	Slow parasitoid development^b^; emergence of parasitoid offspring; host dies.
*Incompatible interactions*		
1	2	**Scenario 1—without non‐reproductive host killing:** No interaction; host survives. **Scenario 2—with non‐reproductive host killing:** Fast parasitoid development^b^, but no emergence of viable parasitoid offspring; host dies.
2	1	

^a^ Outcome under the assumption that parasitoid development completes before the end of the vulnerable developmental period of the host; otherwise, the host survives.

^b^ Developmental rate of generalist (*S* = 3) assumed to be slower than specialists (*S* = 1,2), with the magnitude of the difference being dependent on $\tau_{d}^{P_{3}}$, relative to $\tau_{d}^{P_{1}}\;\text{and}\;\tau_{d}^{P_{2}}$. Compatible interactions do not have an inherent advantage in developmental rate over incompatible interactions.

Parasitoid offspring can only develop if they are compatible with the host (Table 2, Figure 2). This comprises the ability of the parasitoid to utilize the hosts' resources for its own growth, its ability to inactivate hemocytes or regulate the host physiology or behavior to facilitate the development of the parasitoid offspring or its ability to otherwise suppress or evade the immune system of the host. Incompatibility indicates that the parasitoid offspring does not succeed until the end of its development and dies. This would correspond to endo‐parasitoids that come into contact with the internal immune system of the host, such as larval parasitoids attacking *Drosophila* (Rizki & Rizki, 1990). Egg and pupal parasitoids often do not have to deal with cellular immune response (but see Reed et al., 2007; Yang et al., 2019), as eggs and pupal stages mostly rely on their external barrier, such as egg coating and the thickness of the chorion or puparium wall. This may prevent the entire parasitization event from happening but would also constitute a form of incompatibility (Fatouros et al., 2020; Vinson, 1990).

The outcome of an incompatible host–parasitoid interaction differs between scenario 1 and 2 (Table 2). In the model scenario *without* non‐reproductive host killing (scenario 1), either the parasitoid offspring develops when it is compatible with the host, or the host survives the parasitoid attack when they are incompatible. Thus, in this scenario, either the host or the new parasitoid offspring survives the interaction. In the other model scenario *with* non‐reproductive host killing (scenario 2), parasitoid offspring can initially develop in the incompatible hosts to the point that the host is killed, but parasitoid offspring cannot successfully complete development in an incompatible host. Host death can come about due to, for example, damage inflicted by parasitoid attack and/or self‐harm through mounting an immune defense. Hence, when the host is parasitized, it always dies, whether or not it is compatible. Thus, in this scenario, either the host or the parasitoid survives parasitization.

Irrespective of compatibility, the rate of parasitoid development is taken to depend only on the strategy *S* of the parasitoid parent: offspring of a generalist parasitoid are assumed to develop more slowly than the offspring of specialists, such that their expected time of development $\tau_{d}^{P_{3}}$ is larger than for specialists ($\tau_{d}^{P_{1}}\;\text{and}\;\tau_{d}^{P_{2}}$; we assume these to be equal for simplicity) and thus have a lower success rate of completion of development and offspring survival (e.g., Desneux et al., 2009; Raymond et al., 2016). In all cases, their developmental time is drawn from an exponential distribution.

The eventual fate of a parasitized host is determined by the relative timing of its infestation relative to the hosts' sensitive period: if a parasitoid infests the host before it reaches the end of the sensitive period, parasitization will result in killing of the host (Figure 2). As we focused on solitary parasitoids, this event is associated with the emergence of a single new parasitoid individual, unless the host is incompatible (note that incompatible hosts are killed only in model scenario 2, with non‐reproductive host‐killing). When a host has been attacked multiple times by a conspecific parasitoid (superparasitism), the first parasitoid offspring that completes development is decisive for the fate of the host; the offspring of the other parasitoids are always inviable. Finally, when the host reaches the end of the sensitive developmental period before any parasitoid infests it the host survives.

After the outcome of all interactions has been decided, the parasitoid offspring replace the parental generation (such that their total number sets the value of *P* [*t* + 1]), and surviving hosts are collected to determine $H_{1}\left(t+1\right)$ and $H_{2}\left(t+1\right)$, the density of hosts at the start of the next time step.

### 2.5 Genetic assumptions, initial conditions and simulation details

Hymenoptera parasitoids are haplodiploid, but for simplicity and comparability with other host–parasitoid models (e.g., Fellowes & Travis, 2000; Sasaki & Godfray, 1999), host preference and parasitization strategy were each genetically encoded by a single haploid locus, and we assumed reproduction to be clonal. Simulations were initialized with a small initial population of parasitoid individuals, all with S=1 (pre‐adapted to the original host (host 1); incompatible with the invader (host 2) and q=0 (no initial host discrimination). Host population densities were initialized at their equilibrium density in the absence of parasitization ($H_{i}^{*}=K_{i∙}\ln\left(r_{i}\right)$). Genetic variation in host preference and parasitization strategy was introduced by a low rate of mutation (Dieckmann & Law, 1996; Geritz et al., 1997; Metz et al., 1992): mutations in host preference occurred with probability $\mu_{q}$ per reproductive event, and changed the offspring's *q‐*value to $q_{\text{offspring}}=\min\left(1,\max\left(0,q_{\text{parent}}+\Delta q\right)\right)$, where the mutational effect size Δq was sampled from a standard normal distribution; mutations in the parasitization strategy occurred with a low probability $\mu_{S}$ per reproductive event, and changed the tactic from $S_{\text{parent}}$ to $S_{\text{offspring}}$ with probabilities $\mathrm{Pr}\left[S_{\text{parent}}\to S_{\text{offspring}}\right]\;$, given by $\mathrm{Pr}\left[1\to 3\right]=1$, $\mathrm{Pr}\left[2\to 3\right]=1$ and $\mathrm{Pr}\left[3\to 1\right]=\mathrm{Pr}\left[3\to 2\right]=1/2$ (all conditional on the occurrence of a mutation). Accordingly, specialists can mutate to the generalist tactic, which can mutate to either one of the specialist tactics with equal probability, but specialists cannot mutate to become specialized for the other host species in a single mutational step.

The model was implemented as a stochastic individual‐ and event‐based simulation in the programming language C++ (Appendix S1), based on a modification of the Gillespie algorithm for stochastic simulations (Gillespie, 2007). Data produced by the simulation (population densities of hosts and parasitoids; frequencies of parasitization tactics and distribution of host preference) were analyzed in R (version 4.0.1) (R Core Team, 2020). We modeled the two main mechanisms to cope with the evolutionary trap (behavior avoidance or adaptation), by allowing the population to evolve under different mutation probabilities of either parasitization strategy alone or parasitization strategy and host‐species preference and tested how the costs of exerting host preference and the developmental costs of broadening the host‐range influenced the evolution of the parasitoid. These trade‐offs were investigated by altering the host patch search time $\tau_{s}$ and the likelihood of successful infestation by generalist parasitoids, as determined by the parameter $\tau_{d}^{P_{3}}$ (Table 1). We did this under two scenarios: one in which incompatible interaction had no effect on host survival and one in which incompatible interaction resulted in host death (Table 2, Figure 2). Each combination of parameter settings was replicated 30 times and run for 1000 time steps (generations).

## 3 RESULTS

Multiple traits can be involved in host‐range and host adaptation, which might evolve independently in response to selection. We therefore first investigated how genetic variation in parasitization tactic (*S*), the parasitoid's host‐use strategy that determines its physiological compatibility with either of the host species or both, would influence (physiological) host‐range evolution. Next, we also considered genetic variation in host‐preference behavior (*q*), the parasitoid's inherent choice to lay eggs in certain hosts. We did this under different costs by examining the influence of (1) the relative developmental success of generalist parasitoids (determined by different parameter ratios $\tau_{d}^{P_{1}}/\tau_{d}^{P_{3}}$) and (2) parasitoid search time (depending on parameter $\tau_{s}$). Search time may vary depending on environmental conditions: a low search time might reflect a resource rich‐environment in which patches are clustered, whereas a relatively high search time might reflect a resource‐poor environment in which host‐habitat patches are sparse and/or more difficult to locate. The latter conditions imply a higher evolutionary costs of exerting host preference, as the decision to reject a patch would necessitate a large time investment for finding a more suitable patch, compromising the amount of time remaining for reproduction.

Initially, all parasitoids were specialists of host species 1, meaning that exploitation of this host results in one parasitoid offspring and host mortality. Parasitoids were not able to discriminate between patches of host 1 or 2 (no preference, random host patch selection) and lost time exploiting unsuitable host patches. As such, host 2 acted as an “evolutionary trap.” When specialists attempt to exploit an incompatible host, this either had no effect on host survival (model scenario 1), or resulted in host death without parasitoid offspring, that is, non‐reproductive host killing (scenario 2) (Table 2, Figure 2).

### 3.1 Evolution of parasitization tactic

#### 3.1.1 Scenario 1: Incompatible interaction has no effect on host survival

**FIGURE 3:**
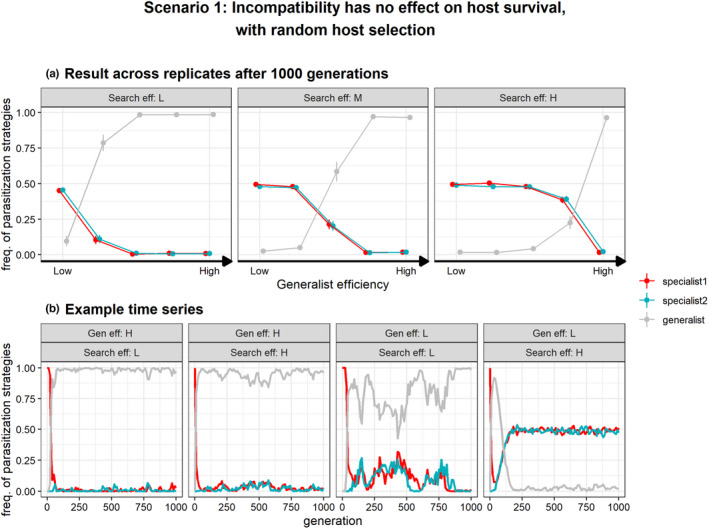
Evolution of parasitization strategy (*S*) with fixed host‐preference (*q* = 0, i.e., random dispersal) under scenario 1 in which incompatible interactions have no effect on host survival. Specialist 1 (red) represents parasitoids able to reproduce on host 1, but incompatible with host 2 and vice‐versa for specialist 2. Generalists (gray) are able to reproduce on both host species but vary in respect to their host use efficiency relative to specialists (‘generalist efficiency’). At generation 0, only specialists of host 1 occur (red) but also attack hosts that do not match their parasitization strategy due to random host searching. They can evolve a generalist strategy (gray) and subsequently mutate to become host use specialist of host 2 (blue). Panel (a) shows relative frequencies of specialist and generalist parasitization strategies (±SE) after 1000 generations (*n* = 30) at various generalist efficiencies and host‐patch search efficiencies (low, $\tau_{s}=11$; medium, $\tau_{s}=7$; high, $\tau_{s}=3$). Hence, which genotype(s) dominate depends on the replicate. Panel (b) shows four examples time series of parasitization strategies at low and high generalist efficiencies ($\tau_{d}^{P_{3}}=45.5,{\;\tau }_{d}^{P_{3}}=42.5\;\text{resp}.$) and low and high host‐patch search efficiencies ($\tau_{s}=11,\tau_{s}=3\;\text{resp}.)$

First, we consider the scenario where an incompatible interaction between a host and parasitoid does not result in host‐killing (scenario 1, Table 2, Figure 2). This can occur for example when the host is unsuitable for development of the parasitoid offspring and the host survives the attack by encapsulation of the parasitoid egg (Kacsoh & Schlenke, 2012; Vinson, 1990). Figure 3 shows example simulations of relative trait values for parasitization strategy over 1000 generations in which host preference is not allowed to evolve and parasitoids exhibit random host patch selection under low and high efficiencies of generalists and host searching. A mutation of parasitization strategy (S) first results in the invasion of a generalist parasitization strategy which is able to overcome the unsuitability of the novel host and thus able to reproduce on host 1 as well as host 2 for reproduction. Note that we assumed that a specialist of host 1 cannot directly shift to the alternative specialist strategy and must first become a generalist by mutation. However, when generalists are less efficient in parasitization (and without non‐reproductive host killing), selection ultimately favors the evolution of a polymorphism of two specialists, after the specialist for host 2 has emerged by mutation from the generalist strategy (Figure 3b). Hence, as expected, low generalist efficiencies result in a consistently higher relative frequency of specialists across replicate simulations (Figure 3a).

Interestingly, the parasitoid search efficiency also influences evolution of specialization. Generalization tended to evolve at low and/or medium search efficiencies while specialization tended to occur at high search efficiencies (Figure 3a). Even at the lowest generalist efficiency, generalists can sometimes invade the population when search efficiency is low as shown in the example timeseries in Figure 3b. Although note that this rarely occurred across replicate simulates under these extreme parameter values (Figure 3a). An explanation is that higher search efficiencies increase the number of host‐patches a parasitoid can visit during its life. This increases the chance of a specialist to find a patch with suitable hosts and give them a competitive advantage even when costs of generalists are relatively low. As such, the search efficiency of the parasitoid can exceed the costs of a narrow host use and facilitate evolution of specialization on the novel host. In contrast, at low/medium search efficiency, generalists have an advantage as they can successfully exploit hosts on every patch they visit, and thus will always find a patch with suitable hosts. Yet, independent of the parasitoid search efficiency and the magnitude of the generalist‐specialist trade‐off, genetic variation in host‐use under scenario 1 consistently allowed parasitoids to adapt to the “trap” host, establishing a novel host–parasitoid relationship (Figure 3a), either by generalization or by specialization. Note that parasitoids with a physiological specialist tactic can still make maladaptive host choices when accepting patches of their unsuitable host due to their random host selection behavior.

#### 3.1.2 Scenario 2: Incompatible interaction results in host‐killing

**FIGURE 4:**
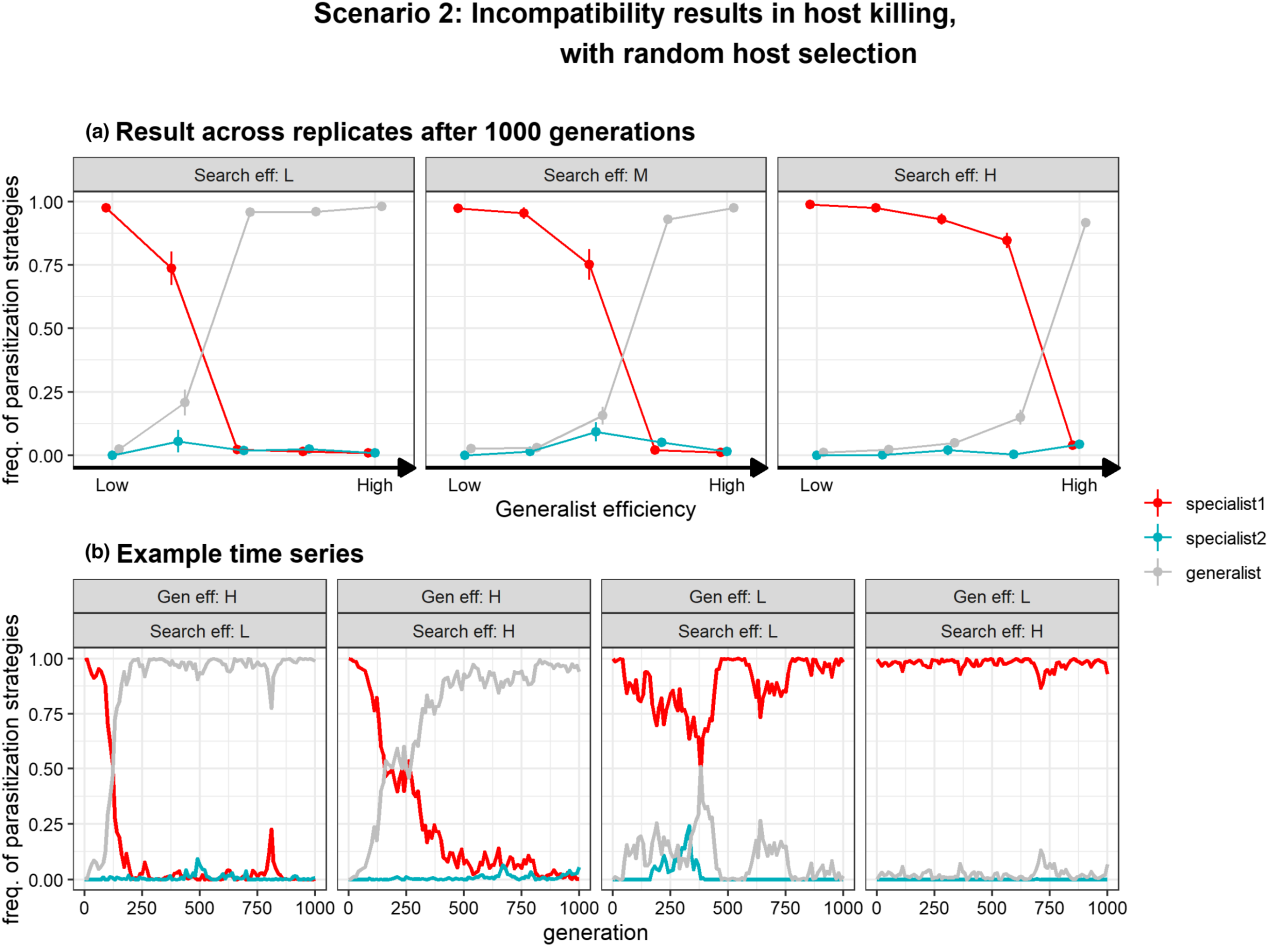
Evolution of parasitization strategy (S) with fixed host‐preference (*q* = 0, i.e., random dispersal) under scenario 2 in which incompatible interactions results in host‐killing. Specialist 1 (red) represents parasitoids able to reproduce on host 1, but are incompatible with host 2 and vice‐versa for specialist 2. Generalists (gray) are able to reproduce on both host species but vary in respect to their host use efficiency relative to specialists (“generalist efficiency”). At generation = 0, only specialists of host 1 occur (red) but also attack hosts that do not match their parasitization strategy. They can evolve a generalist strategy (gray) and subsequently mutate to become host use specialist of host 2 (blue). Panel (a) shows relative frequencies of specialist and generalist parasitization strategies (±SE) after 1000 generations (*n* = 30) at various generalist efficiencies and host‐patch search efficiencies (low, $\tau_{s}=11$; medium, $\tau_{s}=7$; high, $\tau_{s}=3$). Hence, which genotype(s) dominate depends on the replicate. Panel (b) shows four examples time series of parasitization strategies at low and high generalist efficiencies ($\tau_{d}^{P_{3}}=45.5,{\;\tau }_{d}^{P_{3}}=42.5\;\text{resp}.$) and low and high host‐patch search efficiencies ($\tau_{s}=11,\tau_{s}=3\;\text{resp}.$)

Second, we consider the scenario where an incompatible host–parasitoid interaction results in host killing instead of host survival (Scenario 2, Figure 2, Table 2). This might occur when the host dies due to physiological costs; e.g., self‐harm through mounting an immune defense. A similar pattern arises for a subset of conditions: (1) relatively low developmental costs of broadening the physiological host‐range resulted in the evolution of a generalist parasitization tactic and (2) high search efficiencies combined with low efficiency of generalists promoted parasitization specialists (Figure 4). However, in contrast to the simulations without host killing (scenario 1), either specialists of host 1 or generalists dominated after 1000 generations, but specialists of the novel host did not evolve (Figure 4). In other words, non‐reproductive killing of unsuitable hosts can prevent specialists of host 2 to evolve, constraining adaptation and the establishment of novel host–parasitoid relationship. This appeared in particular when parasitoids exhibited a high search efficiency and generalists have relative low host use efficiency (Figure 4), conditions which promoted the evolution of specialists of the unsuitable host under scenario 1. The ability to kill unsuitable hosts allows specialists of host 1 to compete with generalists by reducing the number of available hosts for generalists to exploit hindering the evolution of generalization. Consequently, the evolution of specialization on the (initial) unsuitable host 2 is hampered as well, because specialists of host 2 can only evolve through mutation of the generalist strategy. Hence, non‐reproductive host killing can increase the competitive advantage of specialists over generalists and limit the evolution of specialization on host species 2 resulting in physiological host‐range conservation. This also means that non‐reproductive host killing can be maintained and expressed under these conditions when attacking unsuitable hosts.

### 3.2 Combined evolution of host‐preference and parasitization tactic

We next allowed both parasitization strategy (*S*) and the behavioral trait host preference to evolve (*q*). Host preference relies on individuals rejecting host patches, which is a costly decision if the time needed to find a patch is high. Parasitoids that exhibit an avoidance for either host species can therefore increase their total reproductive output by selecting suitable host‐patches that match their parasitization tactic (performance) and thus lose less time exploiting unsuitable hosts. The evolution of host species preference can however be constrained by the search efficiency of individuals in a given environment as this sets their time budget to find their preferred hosts (Figure 1).

#### 3.2.1 Scenario 1: Incompatible interaction has no effect on host survival

**FIGURE 5:**
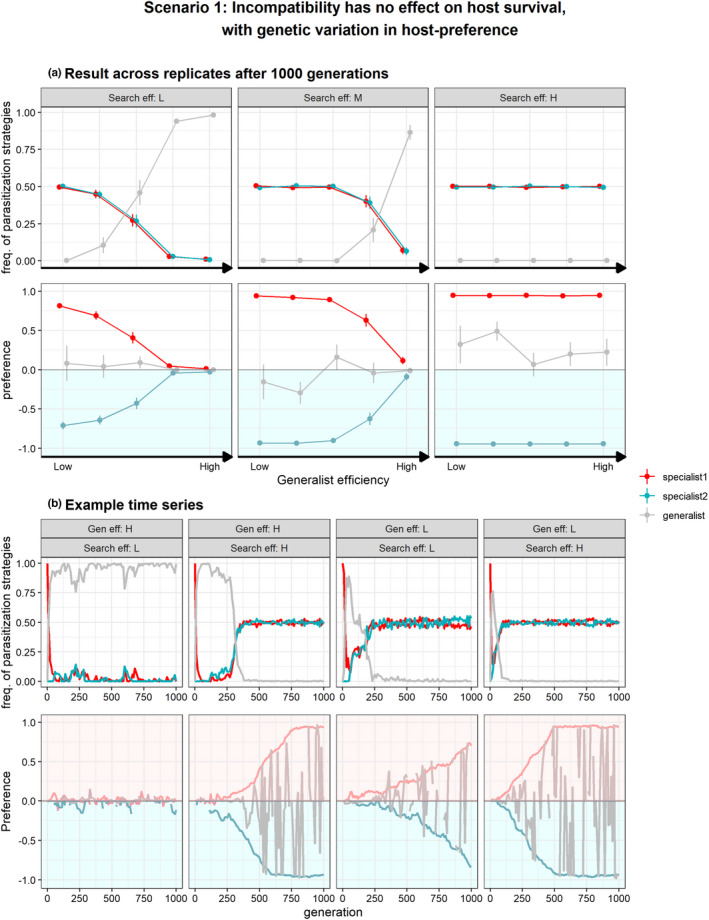
Evolution of both parasitization strategy (S) and host‐preference (*q*) under scenario 1 in which incompatible interactions have no effect on host survival. Specialist 1 (red) represents parasitoids able to reproduce on host 1, but are incompatible with host 2 and vice‐versa for specialist 2. Generalists (gray) are able to reproduce on both host species but vary in respect to their host use efficiency relative to specialists (“generalist efficiency”). At generation = 0, only specialists of host 1 occur (red) and exhibit a random host searching (i.e., no preference, *q =* 0) and thus also attack hosts that do not match their parasitization strategy (host 2). They can evolve a generalist strategy (gray) and subsequently mutate to become host use specialist of host 2 (blue). Preference of 0 indicates random host searching and parasitoids can evolve preference for host‐1 (p > 0) or preference for host 2 (p < 0). Panel (a) shows relative frequencies of specialist and generalist parasitization strategies (±SE) and preference genotypes after 1000 generations (*n* = 30) at various generalist efficiencies and host‐patch search efficiencies (low, $\tau_{s}=11$; medium, $\tau_{s}=7$; high, $\tau_{s}=3$). Hence, which genotype(s) dominate depends on the replicate. Panel (b) shows four examples time series at low and high generalist efficiencies ($\tau_{d}^{P_{3}}=45.5,\tau_{d}^{P_{3}}=42.5\;\text{resp}.$) and low and high host‐patch search efficiencies ($\tau_{s}=11,\tau_{s}=3\;\text{resp}.$)

Figure 5a shows the relative frequency of parasitization tactic and host‐preference after 1000 generations under scenario 1 in which an incompatible interaction has no effect on host survival (Figure 2, Table 2). Figure 5b shows example simulations of relative trait values for both parasitization strategies and host preference over 1000 generations, with various levels of generalist host exploitation efficiency and host searching efficiency. The evolutionary response to the unsuitable host trap was qualitatively similar to the situation with fixed random host selection behavior (Figure 3): consistent adaptation towards the ‘trap’ host among replicates by change in parasitization strategy with a switch from the evolution of a generalist towards specialist tactic with decreasing generalist efficiencies and increasing search efficiencies. However, the conditions for evolution of specialization are relaxed by combined evolution of host preference and parasitization tactic (Figure 5). The evolution of parasitization specialists was facilitated because specialists gain an advantage over generalists by being able to select patches that matches their performance. As such, specialists are able to avoid their unsuitable host (preference ≠ 0) in presence of genetic variation in host‐preference (Figure 5). Only at low host‐search efficiencies and high generalist efficiencies, parasitoids able to reproduce on both hosts were selected, thus resulting in adaptation by host‐range expansion instead of a physiological host‐shift. Low search efficiency increases the time for individuals to find suitable host patches, making it costly to be choosy, promoting random host acceptance. For example, whereas at t ~ 500 parasitoids with high search efficiency evolved as specialists with a behavioral preference for their suitable host, a complete avoidance of their unsuitable hosts generally did not evolve at t = 1000 when search efficiency was low (Figure 5). Low search efficiency can therefore favor random host acceptance (preference = 0) over host avoidance, giving individuals with a generalist parasitization strategy an advantage. Note that host‐preference of generalists highly fluctuates when generalists occur at low frequency and are not evolving (Figure 5a). The erratic pattern is therefore simply the result of the low number of parasitoids with generalist parasitization tactic that stochastically mutated among parental genetic background. In conclusion, genetic variation in host preference (1) promotes parasitoids with a specialist host use strategy and (2) can facilitate the evolution and co‐existence of two distinct specialist strategies in response to the evolutionary trap: one evolving trap avoidance; the other evolving a preference and specialization for the novel host.

#### 3.2.2 Scenario 2: Incompatible interaction results in host‐killing

**FIGURE 6:**
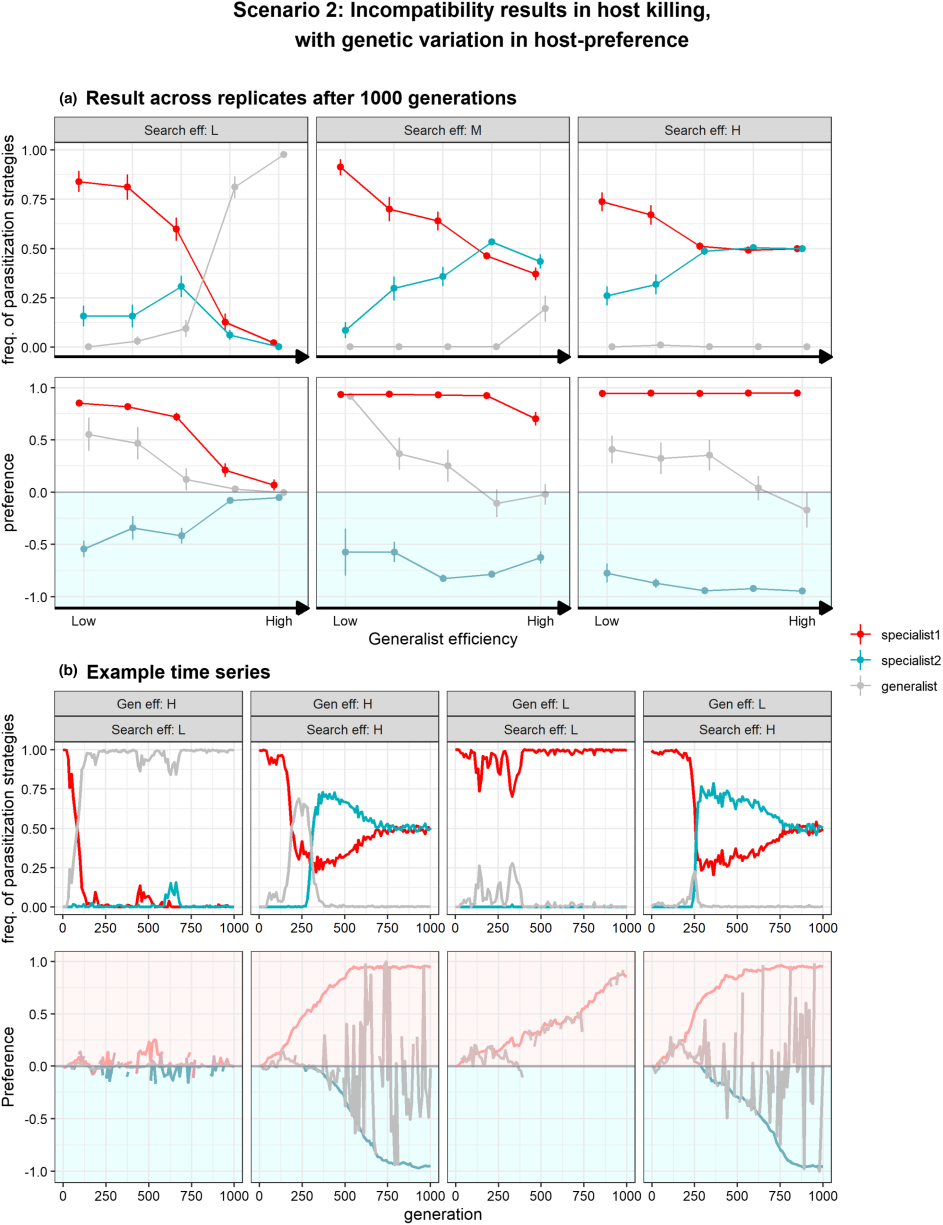
Evolution of both parasitization strategy (S) and host‐preference (q) under scenario 2 in which incompatible interactions result in host killing. Specialist 1 (red) represents parasitoids able to reproduce on host 1, but are incompatible with host 2 and vice‐versa for specialist 2. Generalists (gray) are able to reproduce on both host species but vary in respect to their host use efficiency relative to specialists (“generalist efficiency”). At generation = 0, only specialists of host 1 occur (red) and exhibit a random host searching (i.e., no preference, q = 0) and thus also attack hosts that do not match their parasitization strategy (host 2). They can evolve a generalist strategy (gray) and subsequently mutate to become host use specialist of host 2 (blue). Preference of 0 indicates random host searching and parasitoids can evolve preference for host 1 (p > 0) or preference for host 2 (p < 0). Panel (a) shows relative frequencies of specialist and generalist parasitization strategies (±SE) and preference genotypes after 1000 generations (*n* = 30) at various generalist efficiencies and host‐patch search efficiencies (low, $\tau_{s}=11$; medium, $\tau_{s}=7$; high, $\tau_{s}=3$). Hence, which genotype(s) dominate depends on the replicate. Panel (b) shows four examples time series at low and high generalist efficiencies ($\tau_{d}^{P_{3}}=45.5,\tau_{d}^{P_{3}}=42.5\;\text{resp}.$) and low and high host‐patch search efficiencies ($\tau_{s}=11,\tau_{s}=3\;\text{resp}.)$

As explained above, when parasitization strategy alone was allowed to evolve under scenario 2, the parasitoid's potential to adapt to the unsuitable host was hindered resulting in physiological host‐range conservation (domination of specialists of host 1), remaining a mismatch in host choice and performance (Figure 4). Only when search efficiency was high and costs of being a generalist low, evolution of generalist strategy occurred, although a complete physiological host‐shift did not occur by evolution of specialists of host 2. When non‐reproductive host killing occurred, interestingly, heritable variation in both host preference and performance also changed the evolutionary outcome. In this case, it resulted in evolution of specialists of the ‘trap’ host (host 2) allowing them to co‐occur with specialist of host 1 (Figure 6). In other words, genetic variation in both traits increases the chance of establishment of novel host–parasitoid interaction and allows specialization to the unsuitable host by a complete physiological host‐shift instead of host‐range expansion. This indicates that in the situation of parasitoids killing unsuitable hosts, evolution of avoidance behavior matters. This is because parasitoids with a specialist host use tactic of host 1 can evolve a complete avoidance of the trap; this reduces killing of unsuitable host 2 and removal of resources for parasitoids with a genotype enabling to reproduce on the trap host. Hence, evolution of host‐preference can decrease the strength of competition through non‐reproductive host killing when specialists evolve host‐preference that matches their parasitization strategy, facilitating evolution and persistence of specialists of host 2.

Specialists of host 1 that exhibit non‐reproductive host killing of host 2, however, tend to dominate and constrain physiological host‐range expansion and specialization on the (initial) suboptimal host when search efficiency is low and when generalists exhibit relative high efficiencies (Figure 6). First consider that the individuals' time budget shapes the trade‐off between random dispersal and the optimization of behavior preference with parasitization host use strategy. It follows that when search time constrains evolution of optimal host‐preference, frequent killing of incompatible hosts impairs the evolution and persistence of generalists. Next, as a physiological host‐shift is based on sequential evolution of a generalist to specialist strategy, a high cost of being a generalist further reduces the chance that specialists of host‐2 can appear and persist. Hence, when parasitoids exhibit non‐reproductive host killing, a complete physiological host‐shift is most likely to occur when (1) specialists of host 1 exhibit preference for host 1 reducing intra‐specific competition and (2) when search efficiency is high, reducing the costs of being choosy and allowing a behavioral preference for host 2 to evolve.

## 4 DISCUSSION

The rising number of exotic (invasive) species calls for understanding and predicting not only the—short‐term—ecological impact on native species in the invaded range but also their impact over evolutionary time (Mooney & Cleland, 2001; Schlaepfer et al., 2005; Strauss et al., 2006). Invasive species can act as an evolutionary trap for parasitoids when they are unsuitable for reproduction but indistinguishable from suitable hosts (Schlaepfer et al., 2002; Schlaepfer et al., 2005). Such incompatible interactions reduce parasitoid fitness and results in a mismatch between host choice and offspring survival (performance), that is, “bad motherhood” (Thompson, 1988; Yoon & Read, 2016). Numerous studies have reported a negative relationship between host choice and performance when native parasitoids attack a non‐native host species (Konopka et al., 2018; Martini et al., 2019; Romero et al., 2020). One such example is the solitary koinobiont endo‐parasitoid *L. heterotoma* attacking the invasive *D. suzukii* fruit‐fly. Although this fruit‐fly species is unsuitable for parasitoid offspring development due to its high resistance (Kacsoh & Schlenke, 2012), this incompatible host–parasitoid interaction sometimes results in host mortality (Kruitwagen et al., 2021). Other examples in which parasitoids show low performance in exotic host species include native scelionine egg parasitoids attacking *H. halys*, euphorinae parasitoids attacking adult *H. axyridis* and exoristinae larval parasitoids attacking *C. perspectalis* (Firlej et al., 2012; Konopka et al., 2018; Martini et al., 2019).

Using the *L. heterotoma*–*D. suzukii* system as inspiration, in this study, we explored which conditions could promote parasitoid adaptation through improved performance on the trap host and reduce suboptimal host choices through change in host preference. Whereas trap avoidance would protect parasitoid populations from becoming extinct, adaptation would also enhance their capacity to suppress invasive hosts and thus their value for biological control. Using an individual based model, we show that the outcome of incompatible host–parasitoid interaction matters for the potential of the parasitoid to establish a new viable host–parasitoid relationship with the trap host. While compatible interactions are always fatal for the host (reproductive host killing), we considered that attacking unsuitable hosts either leaves the host unharmed or, on the other extreme, is fatal for the host. The latter often occurs in host–parasitoid systems (e.g., Abram et al., 2016, 2019; Heimpel et al., 2003; Kruitwagen et al., 2021; Liu et al., 2015; Zhou et al., 2019), but little is known about its influence on ecological and evolutionary processes. We found that non‐reproductive host killing can hamper adaptation to the trap in conditions which in fact promoted host‐range evolution in parasitoids when they were not able to kill unsuitable hosts. Moreover, we show that the establishment of a novel host–parasitoid relationship does not necessarily have to occur at the behavioral level through changing host‐preference behavior, as a physiological strategy can evolve that enables reproduction on the trap. However, evolved behavioral preference promotes adaptation to the trap by a complete host‐shift and allows parasitoids to evolve avoidance of their unsuitable host, minimizing suboptimal host choices.

### 4.1 Non‐reproductive host killing constrains evolution

Even when parasitoids are not able to exploit hosts for reproduction, their attack can still reduce host survival rate due to, for example, wounding or immune defense costs (Abram et al., 2016, 2019; Kruitwagen et al., 2021). Previous empirical and modeling studies showed that magnitude of non‐reproductive host killing can influence host–parasitoid population dynamics via direct and indirect interactions (Heimpel et al., 2003; Kaser et al., 2018). We found that when incompatible host interactions result in host killing it also influences the ability of the parasitoid to adapt to a trap host. This adaptation entails establishing a novel compatible host–parasitoid relationship. While adaptation to the trap consistently occurred when parasitoids did not affect survival of their unsuitable host, adaptation can be constrained when parasitoid attack is destructive for the host. This is because the removal of resources through non‐reproductive host killing increases the strength of competition with (generalist or specialist) genotypes compatible with the trap host and can therefore hamper their evolution. In fact, when parasitoids exhibit non‐reproductive host killing of unsuitable hosts, their strategy appeared to be an “evolutionary dead end”: parasitoids remained attacking the trap without changing their host use tactic to include the host into their repertoire. This occurred in particular when (1) specialist parasitoids were more efficient in parasitizing their host compared to genotypes with a generalist host use strategy and (2) the encounter rate with the trap host was high as a result of a high efficiency to find host patches and random‐host selection behavior. This makes sense as a high encounter rate increases the parasitoid impact on the host population size and thus their competitive advantage.

We assumed a fixed high intrinsic growth rate and equal number of host‐patches of each host to support the parasitoid population. In nature however, variation in environmental factors such as climate conditions and resource quality and quantity might cause temporal variation in the hosts' reproduction potential and carrying capacity (e.g., Behrman et al., 2015; Lue et al., 2018). Moreover, host‐species population dynamics might be altered when hosts share (some) resources resulting in inter‐specific competition. As the host community structure influences the magnitude and direction of selection of the parasitoid, future modeling studies could focus on how more complex host population dynamics influence the role of non‐reproductive host killing on the evolutionary trajectory of the parasitoid. Some relevant factors that could be included are stochastic changes in reproductive potential of the hosts, the relative number of host patches and inter‐specific competition between the host species. It would therefore be very interesting to implement field survey data to increase the predictive value of future models.

### 4.2 Bad motherhood and evolution of host‐preference

The “jack of all trades are masters of none” assumption postulates a generalist‐specialist trade‐off (Futuyma & Moreno, 1988). Indeed, we found that when generalists bear significant costs, it is more profitable to specialize to the trap by adopting a specialist host‐use tactic. Although this can result in a new compatible host–parasitoid relationship, when parasitoids lose their ability to use their original host but do not (yet) evolve behavioral avoidance of these original hosts, this can still result in suboptimal host choices, that is, their physiological and behavioral host‐range do not match. We found that ‘bad motherhood’ can be lifted when parasitoids also exhibit genetic variation in host preference. Joint evolution of host‐preference and performance (host use tactic) enables parasitoids to maximize their reproduction by reducing their time spend in suboptimal host patches, resulting in host choices that match their performance. This is relevant as traits that influence host localization and preference in various parasitoid species are found to be influenced by genetics (e.g., Desjardins et al., 2010; Dubuffet et al., 2006; Hopper et al., 2019; Rolff & Kraaijeveld, 2001). The model showed that evolution of host preference promotes evolution of host‐use specialists by increasing their advantage over generalists and reducing suboptimal host decisions. Likewise, resource‐consumer studies showed that evolution of habitat choice qualitatively changes adaptation by promoting specialist over generalist and allowing different specialists to coexists (Ravigné et al., 2009; Rueffler et al., 2007).

Despite genetic variation in host‐preference, we found that ‘bad motherhood’ can still persist when parasitoid efficiency to locate host patches is low. According to optimal foraging models for time‐limited parasitoids, females should maximize their host encounter rate to maximize their reproductive output (Comins & Hassell, 1979; Wajnberg, 2006). Our findings are in agreement with other studies (McNamara et al. (1993); e.g., Barrette et al. (2010)) that found that optimal foraging may include acceptance of less profitable hosts/prey to maximize reproductive output when travel time between patches is long. This however might not be the case for egg‐limited parasitoids (such as synovigenic insects) as they are predicted to maximize the quality of hosts they accept rather than their host encounter rate (Fletcher et al., 1994; Minkenberg et al., 1992). A thorough understanding of possible outcomes under egg‐limitation would require a similar modeling approach as outlined in this paper.

### 4.3 Genetic assumptions and evolutionary trajectory

We assumed haploid genotypes with two single unlinked loci determining host use and host preference with clonal inheritance. It has to be considered however that many parasitoids reproduce sexually through haplodiploidy (arrhenotoky) meaning that unfertilized eggs arise through meiosis and develop into males, and females arise after mating through fusion of two gametes and are diploid (Godfray & Cook, 1997; Heimpel & De Boer, 2008). Moreover, recombination between traits can occur in sexually reproducing parasitoid females at a frequency that seems to be similar to diploid higher eukaryotes (Beukeboom et al., 2010; Niehuis et al., 2010). Consequently, in nature, this may retard/prevent adaptation by specialization under joint evolution of host preference and parasitization strategy through continuous recombination between genotypes (Doebeli & Dieckmann, 2000; Felsenstein, 1981) or may speed up selection by bringing together the two traits (Felsenstein, 1974; Marais & Charlesworth, 2003) depending on, for example, recombination frequency and population size. As our model does not allow for recombination, a similar outcome of the model would be observed if traits interact via epistatic or pleiotropic effects in a sexual reproducing parasitoid species with multilocus genetics, such as when the expression of host preference would be developmentally coupled to the expression of parasitization strategy. Alternatively, matching host‐preference with parasitization performance might arise through simultaneous but independent selection on both traits. In fact, the latter seems more likely as empirical studies indicate that physiological traits have no pleiotropic effect on behavior (host choice) in parasitoids and may therefore evolve parallel to each other (Dubuffet et al., 2006; Rolff & Kraaijeveld, 2001).

Another factor to keep in mind is that both host‐preference and performance are often considered to be complex behavioral and physiological traits (Vinson, 1998; Vinson & Iwantsch, 1980), potentially controlled by multiple (linked) loci (Desjardins et al., 2010; Hawthorne & Via, 2001; Huang et al., 2021; Werren et al., 2010). Moreover, studies show that behavior and physiological traits generally have low heritability (Dochtermann et al., 2019; Kruitwagen et al., 2021; Mousseau & Roff, 1987; Stirling et al., 2002), suggesting that they are also influenced by variation in environmental conditions. For example, we found in a previous study that heritability of attack rate and non‐reproductive host killing in *L. heterotoma* with regard to the invasive *D. suzukii* host is *h* ^2^ = 0.2 (Kruitwagen et al., 2021). Hence, these genetic factors might change the evolutionary trajectory by altering the rate of evolution and/or which traits are selected (Fellowes & Travis, 2000; Kawecki, 1998). For instance, on the one extreme, lack of evolution in host‐preference under expression of non‐reproductive host killing may seriously constrain evolution of specialization as we have shown in our model with universal host acceptance (random host searching).

Here we assumed that non‐heritable factors influence the length of the vulnerable period in which hosts can be parasitized. However, hosts could also evolve counter resistance to parasitoid attack, which can consequently result in a co‐evolutionary arms race in virulence (degree of harm) and resistance (Fellowes & Travis, 2000; Kawecki, 1998; Sasaki & Godfray, 1999). Such change in level of virulence might come about through an increase in venom production resulting in a higher incidence of (non‐)reproductive host killing (Cavigliasso et al., 2019; Colinet et al., 2010; Poirié et al., 2009). An interesting next step would therefore be to incorporate parasitoid virulence and host resistance in this model to further investigate the evolutionary response of the parasitoid under different outcomes of host–parasitoid compatibilities.

## 5 CONCLUSION

This model together with previous studies shows that genetics, behavioral, and physiological factors can constrain parasitoids to evolve matching of host‐preference and host‐performance explaining why parasitoids' host selection behavior is not always in agreement with their physiological host use capacity and thus results in a discrepancy in their behavioral and physiological host‐range (e.g., Cronin et al., 2001; Thompson, 1988). This study adds that non‐reproductive host killing is important for the evolutionary host–parasitoid dynamics and outcome in the situation of a novel unsuitable host species. Moreover, our study underlines that not only the parasitoid genetics should be studied, but also the parasitoid behavior, in particular the magnitude of non‐reproductive host killing and host‐finding ability, in their natural environment to predict whether and how parasitoids might adapt to the trap. This together with insight in time allocation “decisions” of a particular host–parasitoid system is in fact of great value for pest control: time allocated to traveling relative to residency on different patches will determine their success to suppress pest populations (Mills & Wajnberg, 2008; Wajnberg et al., 2016). Hence, whether and how parasitoids evolve influences their host‐range, and thus their ability to regulate novel hosts and their value for biological control.

## AUTHOR CONTRIBUTIONS

**Astrid Kruitwagen:** Conceptualization (lead); formal analysis (lead); methodology (equal); writing – original draft (lead); writing – review and editing (equal). **Leo W. Beukeboom:** Conceptualization (equal); methodology (equal); writing – review and editing (equal). **Bregje Wertheim:** Conceptualization (equal); methodology (equal); writing – review and editing (equal). **G. Sander van Doorn:** Conceptualization (equal); methodology (lead); writing – review and editing (equal).

## CONFLICT OF INTEREST

The authors declare no conflict of interest. The funders had no role in the design of the study; in the collection, analyses, or interpretation of data; in the writing of the manuscript, or in the decision to publish the results.

### OPEN RESEARCH BADGES

This article has earned an Open Data badge for making publicly available the digitally‐shareable data necessary to reproduce the reported results. The data is available at: https://doi.org/10.5061/dryad.31zcrjdp6. The model code can be found in the Appendix S1.

## Supporting information


Appendix S1

## Data Availability

Please see Dryad, https://doi.org/10.5061/dryad.31zcrjdp6.
