# Supplementary material for: Evolution of parasitoid host preference and performance in response to an invasive host acting as evolutionary trap
Source: Ecol Evol. 2022 Jul 4;12(7):e9030. doi: 10.1002/ece3.9030 (PMC9251845; doi:10.1002/ece3.9030)
Supplement: Supplementary file 1 — Appendix S1 [file ECE3-12-e9030-s001.pdf]

```
/*=============================================================================================
                                        APPENDIX S1
===============================================================================================
C++ code accompanying:

        Evolution of parasitoid host preference and performance in response to an
        invasive host acting as an evolutionary trap,
        by: Astrid Kruitwagen, Leo W. Beukeboom, Bregje Wertheim and G. Sander van Doorn
        published in: Ecology and Evolution (2022).

Code developed by:
        G. Sander van Doorn
        Groningen Institute for Evolutionary Life Sciences
        University of Groningen
        the Netherlands

Program version
        01/06/2022  :   final version, archived with publication

Instructions for compiling and running the program

    Versions of this program were compiled and run on Windows and Mac, using
    Visual Studio Code and XCode version 13.4. The code listed below was split
    over two .cpp files (main.cpp and random.cpp) and one header file (random.h).

    The program accepts a single optional program argument, that will be interpreted
    as the name of a .txt file containing parameter values
    (see example file parameters.txt below). If no program argument is provided, a
    simulation will be run using default parameter values specified in main.cpp.

    Output from the simulation data is stored in a comma separated data file, that can
    be imported in R for data visualisation and data processing.

=============================================================================================*/

/*=============================================================================================
                                        FILE MAIN.CPP
=============================================================================================*/

#include <iostream>
#include <fstream>
#include <sstream>
#include <array>
#include <vector>
#include <queue>
#include <algorithm>
#include <cmath>
#include <cstdlib>
#include <exception>
#include "random.h"

/*=============================================================================================
                Parameters and default values
=============================================================================================*/

// host population dynamics
double rHost1 = 5.0;            // loss of stability at ln(r) > 2 <-> r > 7.38906
double rHost2 = 5.0;
double alphaHost1 = 0.01;       // K = 1/alpha * ln(r)
double alphaHost2 = 0.01;
double tMatureHost = 10.0;

// parasitoid life cycle
bool nonreproductiveHostKilling = false;
double tLocatePatch = 1.0;
double tAbandonPatch = 0.1;
double tLocateHost = 0.1;
double tRecover = 1.0;
```

```
double tMatureParasitoid = 10.0;

// selection and mutation
double generalistEfficiency = 1.0;
double sigma = 0.01;
double mu = 0.01;

// simulation parameters
size_t parasitoidPopSize = 10u;
size_t maxIt = 100u;
size_t dataIt = 1u;
const size_t nHostPatches1 = 20u; // fixed at compile-time
const size_t nHostPatches2 = 20u; // fixed at compile-time
unsigned int rseed;

/*============================================================================================
                Class definitions
============================================================================================*/

class HostPatch;

class Parasitoid {
public:
    friend struct EventComp;
    friend class HostPatch;
    Parasitoid();
    Parasitoid(Parasitoid const * const);
    void locatePatch();
    bool exploitPatch();
    double time() const {return t;}
    void getData(std::vector<size_t>&, std::vector<double>&, std::vector<double>&) const;
private:
    HostPatch *hostPatch;
    double t, delta;
    size_t strategy;
    bool killHost;
};

class HostPatch
{
public:
    friend class Parasitoid;
    struct Host {
        Host() : parasitoid(nullptr) {
            deadline = std::exponential_distribution<double>(1.0 / tMatureHost) (rnd::rng);
            if(deadline > lastDeadline) lastDeadline = deadline;
        }
        Parasitoid const * parasitoid;
        double deadline;
        static double lastDeadline;
    };
    HostPatch() : n(0u) {}
    void refill(const size_t);
    void collectOffspring(std::vector<Parasitoid *>&, size_t&, std::array<size_t, 3u> &,
     std::array<size_t, 3u> &, const size_t);
private:
    size_t n;
    std::vector<Host> hosts;
    static size_t nHostTotal;
};

struct EventComp {
    bool operator() (Parasitoid const * const lhs, Parasitoid const * const rhs) const
    {return lhs->t > rhs->t;}
};

typedef std::priority_queue<Parasitoid *, std::vector<Parasitoid *>, EventComp> ParasitoidQueue;
```

```
/*============================================================================
                Main program
============================================================================*/

std::array<HostPatch, nHostPatches1> hostPopulation1;
std::array<HostPatch, nHostPatches2> hostPopulation2;

ParasitoidQueue parasitoidPopulation;

int main(int argc, char * argv[]) {
    void readParameters(const std::string&);
    void validateParameters();
    try {
        // set parameter values
        if(argc == 1) {
            rseed = rnd::set_seed(); // use default parameters values and use clock to set
             random seed
        }
        else if(argc == 2)
            readParameters(argv[1]);
        else throw std::runtime_error("invalid number of program arguments in main()");
        validateParameters();

        // open data file
        std::clog << "** preparing data file **\n";
        std::ostringstream filename;
        filename << "data_" << rseed << ".csv";
        std::ofstream dataFile(filename.str().c_str());
        if(!dataFile.is_open()) throw std::runtime_error("unable to open output file " +
         filename.str());
        const char sep = ',';

        // store parameters in datafile
        dataFile    << "*** Parameter values ***\n\n"
                    << "r_host1" << sep << rHost1 << '\n'
                    << "alpha_host1" << sep << alphaHost1 << '\n'
                    << "r_host2" << sep << rHost2 << '\n'
                    << "alpha_host2" << sep << alphaHost2 << '\n'
                    << "t_mature_host" << sep << tMatureHost << '\n'
                    << "t_locate_patch" << sep <<  tLocatePatch<< '\n'
                    << "t_abandon_patch" << sep <<  tAbandonPatch<< '\n'
                    << "t_locate_host" << sep <<  tLocateHost<< '\n'
                    << "t_recover" << sep <<  tRecover<< '\n'
                    << "t_mature_parasitoid" << sep <<  tMatureParasitoid<< '\n'
                    << "generalistEfficiency" << sep <<  generalistEfficiency<< '\n'
                    << "nonreproductive_host_killing" << sep <<  nonreproductiveHostKilling <<
                     '\n'
                    << "mutation_rate" << sep <<  mu<< '\n'
                    << "mutation_step" << sep <<  sigma<< '\n'
                    << "initial_parasitoid_population_size" << sep <<  parasitoidPopSize<< '\n'
                    << "number_of_iterations" << sep <<  maxIt<< '\n'
                    << "store_data_interval" << sep <<  dataIt<< '\n'
                    << "random_seed" << sep <<  rseed << '\n';

        // write datafile header
        dataFile    << "*** Simulation data ***\n\n"
                    << "t" << sep
                        << "H1" << sep << "surviving" << sep << "parasitised" << sep << sep <<
                         sep << sep << "killed" << sep << sep << sep << sep
                        << "H2" << sep << "surviving" << sep << "parasitised" << sep << sep <<
                         sep << sep << "killed" << sep << sep << sep << sep
                        << "P1" << sep << "avg(delta)" << sep << "stdev(delta)" << sep
                        << "P2" << sep << "avg(delta)" << sep << "stdev(delta)" << sep
                        << "P3" << sep << "avg(delta)" << sep << "stdev(delta)" << sep
                        << "Ptot" << sep << "avg(delta)" << sep << "stdev(delta)\n";

        // create initial parasitoid population
        std::clog << "** initialising simulation **\n";
```

```
std::vector<Parasitoid *> parasitoidOffspring(parasitoidPopSize);
for(int i = 0u; i < parasitoidPopSize; ++i) {
    parasitoidOffspring[i] = new Parasitoid;
}

// fill Host patches
HostPatch::Host::lastDeadline = 0.0;
const size_t KHost1 = rHost1 > 1.0 ?
    static_cast<size_t>(log(rHost1) / (alphaHost1 * nHostPatches1)) : 0u;
const size_t KHost2 = rHost2 > 1.0 ?
    static_cast<size_t>(log(rHost2) / (alphaHost2 * nHostPatches2)) : 0u;
for(HostPatch &patch : hostPopulation1) patch.refill(KHost1);
for(HostPatch &patch : hostPopulation2) patch.refill(KHost2);

size_t initialHost1 = KHost1 * nHostPatches1, initialHost2 = KHost2 * nHostPatches2;

std::clog << "** starting simulation loop **\n";
for(size_t it = 0u; it < maxIt; ++it) {

    // replace parasitoids by offspring
    parasitoidPopulation = ParasitoidQueue(parasitoidOffspring.begin(),
     parasitoidOffspring.end());
    parasitoidOffspring.clear();

    // model host-parasitoid interactions
    if(parasitoidPopulation.empty()) {
        std::clog << "iteration " << it << " : parasitoid extinct\n";
        it = maxIt;
    }
    else
        for(;;) {

            Parasitoid * focal = parasitoidPopulation.top();
            parasitoidPopulation.pop();

            if(focal->time() > HostPatch::Host::lastDeadline || focal->exploitPatch()
             == false) {
                parasitoidPopulation.push(focal);
                break;
            }
            else parasitoidPopulation.push(focal);
        }

    // compute host growth rate and collect parasitoid offspring
    HostPatch::Host::lastDeadline = 0.0;
    size_t survivingHost1 = 0u;
    std::array<size_t, 3u> parasitisedHost1, killedHost1;
    parasitisedHost1[0u] = parasitisedHost1[1u] = parasitisedHost1[2u] = 0u;
    killedHost1[0u] = killedHost1[1u] = killedHost1[2u] = 0u;
    for(HostPatch &patch : hostPopulation1)
        patch.collectOffspring(parasitoidOffspring, survivingHost1, parasitisedHost1,
         killedHost1, 1u);
    const size_t aux0 = parasitoidOffspring.size();
    const double lambda1 = rHost1 * survivingHost1 * exp(-alphaHost1 * survivingHost1)
     / nHostPatches1;
    size_t survivingHost2 = 0u;
    std::array<size_t, 3u> parasitisedHost2, killedHost2;
    parasitisedHost2[0u] = parasitisedHost2[1u] = parasitisedHost2[2u] = 0u;
    killedHost2[0u] = killedHost2[1u] = killedHost2[2u] = 0u;
    for(HostPatch &patch : hostPopulation2)
        patch.collectOffspring(parasitoidOffspring, survivingHost2, parasitisedHost2,
         killedHost2, 2u);
    const double lambda2 = rHost2 * survivingHost2 * exp(-alphaHost2 * survivingHost2)
     / nHostPatches2;

    // store host data
    if(it % dataIt == 0u) {
        dataFile << it << sep;
```

```
        std::cout << "t = " << it << ":\n";
        if(initialHost1) {
            const double aux1 = survivingHost1 * 1.0 / initialHost1;
            const double aux2 = aux0 * 1.0 / initialHost1;
            const double aux3 = (initialHost1 - survivingHost1 - aux0) * 1.0 /
             initialHost1;
            dataFile << initialHost1 << sep << aux1 << sep << aux2 << sep;
            for(size_t x : parasitisedHost1) dataFile << x * 1.0 / initialHost1 << sep;
            dataFile << aux3 << sep;
            for(size_t x : killedHost1) dataFile << x * 1.0 / initialHost1 << sep;
        }
        else {
            dataFile << initialHost1;
            for(int k = 0; k < 10u; ++k) dataFile << sep;
        }
        if(initialHost2) {
            const double aux4 = survivingHost2 * 1.0 / initialHost2;
            const double aux5 = (parasitoidOffspring.size() - aux0) * 1.0 /
             initialHost2;
            const double aux6 = (initialHost2 - survivingHost2 -
             parasitoidOffspring.size() + aux0) * 1.0 / initialHost2;
            dataFile << initialHost2 << sep << aux4 << sep << aux5 << sep;
            for(size_t x : parasitisedHost2) dataFile << x * 1.0 / initialHost2 << sep;
            dataFile << aux6 << sep;
            for(size_t x : killedHost2) dataFile << x * 1.0 / initialHost2 << sep;
        }
        else {
            dataFile << initialHost2;
            for(int k = 0; k < 10u; ++k) dataFile << sep;
        }
    }

    // refill host patches
    initialHost1 = 0u;
    for(HostPatch &patch : hostPopulation1) {
        const size_t k = lambda1 > 0.0 ?
         std::poisson_distribution<size_t>(lambda1)(rnd::rng) : 0u;
        patch.refill(k);
        initialHost1 += k;
    }
    initialHost2 = 0u;
    for(HostPatch &patch : hostPopulation2) {
        const size_t k = lambda2 > 0.0 ?
         std::poisson_distribution<size_t>(lambda2)(rnd::rng) : 0u;
        patch.refill(k);
        initialHost2 += k;
    }

    // collect and store parasitoid data
    if(it % dataIt == 0u) {
        const size_t n = parasitoidOffspring.size();
        std::cout << "\tH1    = " << initialHost1
        << "\tH2    = " << initialHost2
        << "\n\tPtot  = " << n << '\n';
        if(n) {
            std::vector<size_t> counts {0u, 0u, 0u};
            std::vector<double> sumd(3u, 0.0), sumdd(3u, 0.0);

            for(Parasitoid const * parasitoid : parasitoidOffspring)
                parasitoid->getData(counts, sumd, sumdd);

            double avgd = 0.0, stdevd = 0.0;
            for(size_t i = 0u; i < 3u; ++i) {
                if(counts[i]) {
                    avgd += sumd[i];
                    stdevd += sumdd[i];
                    sumd[i] /= counts[i];
                    sumdd[i] = counts[i] > 1u ?
```

```
                            sqrt((sumdd[i] - counts[i] * sumd[i] * sumd[i]) / (counts[i] - 1u))
                             : 0.0;
                            dataFile << counts[i] * 1.0 / n << sep
                            << sumd[i] << sep
                            << sumdd[i] << sep;
                            std::cout << "\t\tP" << i + 1u << "    = " << counts[i] << ";
                             delta" << i + 1u << " = " << sumd[i] << "+/-" << sumdd[i] << '\n';
                        }
                        else dataFile << counts[i] * 1.0 / n << sep << sep << sep;
                    }
                    avgd /= n;
                    stdevd = n > 1u ? sqrt((stdevd - n * avgd * avgd) / (n - 1u)) : 0.0;
                    dataFile << n << sep << avgd << sep << stdevd << '\n';

                }
                else dataFile << n << sep << sep << sep << '\n';
            }

            // remove adult population
            while(!parasitoidPopulation.empty()) {
                Parasitoid * focal = parasitoidPopulation.top();
                delete focal;
                parasitoidPopulation.pop();
            }
        }
        std::clog << "** simulation complete **\n";
        dataFile.close();
    }
    catch(std::exception &err)
    {
        std::cerr << "fatal error : " << err.what() << '\n';
        std::exit(EXIT_FAILURE);
    }
    return EXIT_SUCCESS;
}

/*============================================================================================
                Other functions
============================================================================================*/

template <class T>
bool read(const std::string &str, const std::string &name, T &par, std::ifstream &ifs)
{
    if(str == name) {
        ifs >> par;
        std::clog << "parameter " << str << " set to " << par << '\n';
        return true;
    }
    else return false;
}

void readParameters(const std::string& filename)
{
    std::clog << "** reading parameters from file " << filename << " **\n";

    //open parameter file
    std::ifstream ifs(filename);
    if(!ifs.is_open())
        throw std::runtime_error("unable to open parameter file in readParameters()");

    std::string str;

    ifs >> str;
    if(str == "rng_seed_clock") rseed = rnd::set_seed();
    else if(str == "rng_seed_user") {
        ifs >> rseed;
        rnd::set_seed(rseed);
    }
```

```
    else throw std::logic_error("\'rng_seed_clock\' or \'rng_seed_user <arg>\' expected at
     first line of parameterfile\n");
    while(ifs >> str) {
        if(read(str, "r_host1", rHost1, ifs));
        else if(read(str, "alpha_host1", alphaHost1, ifs));
        else if(read(str, "r_host2", rHost2, ifs));
        else if(read(str, "alpha_host2", alphaHost2, ifs));
        else if(read(str, "t_mature_host", tMatureHost, ifs));
        else if(read(str, "t_locate_patch", tLocatePatch, ifs));
        else if(read(str, "t_abandon_patch", tAbandonPatch, ifs));
        else if(read(str, "t_locate_host", tLocateHost, ifs));
        else if(read(str, "t_recover", tRecover, ifs));
        else if(read(str, "t_mature_parasitoid", tMatureParasitoid, ifs));
        else if(read(str, "generalist_efficiency", generalistEfficiency, ifs));
        else if(read(str, "nonreproductive_host_killing", nonreproductiveHostKilling, ifs));
        else if(read(str, "mutation_rate", mu, ifs));
        else if(read(str, "mutation_step", sigma, ifs));
        else if(read(str, "initial_parasitoid_population_size", parasitoidPopSize, ifs));
        else if(read(str, "number_of_iterations", maxIt, ifs));
        else if(read(str, "store_data_interval", dataIt, ifs));
        else throw std::runtime_error("unknown parameter " + str);
    }
    std::clog << "** parameters were read in successfully **\n";
}

void validateParameters()
{
    if(rHost1 < 0.0) throw std::runtime_error("invalid value for parameter rHost1");
    if(rHost2 < 0.0) throw std::runtime_error("invalid value for parameter rHost2");
    if(alphaHost1 <= 0.0) throw std::runtime_error("invalid value for parameter alphaHost1");
    if(alphaHost2 <= 0.0) throw std::runtime_error("invalid value for parameter alphaHost2");
    if(tMatureHost <= 0.0) throw std::runtime_error("invalid value for parameter tMatureHost");
    if(tLocatePatch <= 0.0) throw std::runtime_error("invalid value for parameter
     tLocatePatch");
    if(tAbandonPatch <= 0.0) throw std::runtime_error("invalid value for parameter
     tAbandonPatch");
    if(tLocateHost <= 0.0) throw std::runtime_error("invalid value for parameter tLocateHost");
    if(tRecover <= 0.0) throw std::runtime_error("invalid value for parameter tRecover");
    if(tMatureParasitoid <= 0.0) throw std::runtime_error("invalid value for parameter
     tMatureParasitoid");
    if(generalistEfficiency <= 0.0) throw std::runtime_error("invalid value for parameter
     generalistEfficiency");
    if(sigma < 0.0) throw std::runtime_error("invalid value for parameter sigma");
    if(mu < 0.0) throw std::runtime_error("invalid value for parameter mu");
}

/*============================================================================================
                Implementation of the class Parasitoid
============================================================================================*/

Parasitoid::Parasitoid() : hostPatch (nullptr), killHost(false), t(0.0), delta(0.0),
 strategy(1u)
// default constructor; called at initialisation
{
    locatePatch();
}

Parasitoid::Parasitoid(Parasitoid const * const parent) : hostPatch (nullptr), killHost(false),
 t(0.0)
// alternative constructor; implements reproduction with mutation
{
    // inheritance of host preference
    const double epsilon = 1.0e-9;
    delta = parent->delta + sigma * rnd::normal(0.0, 1.0);
    if(delta < -1.0 + epsilon) delta = -1.0 + epsilon; // ensure delta >= -1 + epsilon
    if(delta > +1.0 - epsilon) delta = +1.0 - epsilon; // ensure delta <= +1 - epsilon

    // inheritance of parasitisation strategy
```

```
    if(rnd::uniform() < mu) {
        if(parent->strategy == 3u) strategy = rnd::bernoulli() ? 1u : 2u;
        else strategy = 3u;
    }
    else strategy =  parent->strategy;

    locatePatch();
}

void Parasitoid::locatePatch()
// select a patch
{
    // reject host 2 with probability p
    const double p1 = std::min(1.0, 1.0 + delta);
    const double p2 = std::min(1.0, 1.0 - delta);
    const double pAcceptPatch = (p1 * nHostPatches1 + p2 * nHostPatches2) / (nHostPatches1 +
     nHostPatches2);
    if(pAcceptPatch <= 0.0) throw std::logic_error("no acceptable patch for individual in
     Parasitoid::locatePatch()");
    size_t nVisitPatch = pAcceptPatch < 1.0 ? 1u +
     std::geometric_distribution<size_t>(pAcceptPatch)(rnd::rng) : 1u;

    // sample time and patch
    t += std::gamma_distribution<double>(nVisitPatch, tLocatePatch)(rnd::rng);
    if(rnd::uniform() < p1 * nHostPatches1 / (p1 * nHostPatches1 + p2 * nHostPatches2)) {
        hostPatch =  &(hostPopulation1[rnd::random_int(nHostPatches1)]);
        killHost = nonreproductiveHostKilling || (strategy & 1u);
    }
    else {
        hostPatch = &(hostPopulation2[rnd::random_int(nHostPatches2)]);
        killHost = nonreproductiveHostKilling || (strategy & 2u);
    }
}

bool Parasitoid::exploitPatch()
// interact with hosts within a patch
{
    // update host state
    for(size_t i = 0u; i < hostPatch->n;) {

        if(t > hostPatch->hosts[i].deadline) {
            --hostPatch->n;
            std::swap(hostPatch->hosts[i], hostPatch->hosts[hostPatch->n]);
            --HostPatch::nHostTotal;
        }
        else ++i;
    }

    // check for end of season or empty patch
    size_t n = hostPatch->n;
    double tQuit = tAbandonPatch;
    if(!HostPatch::nHostTotal) return false;
    else if(n > 0u) {
        // generate random sequence of encounters
        std::vector<size_t> seq(n);
        std::vector<double> wt(n);
        std::exponential_distribution<double> waitingTime(1.0 / tLocateHost);
        seq[0u] = 0u;
        wt[0u] = waitingTime(rnd::rng);
        tQuit = wt[0u] + waitingTime(rnd::rng); // = second encounter with host
        for(size_t i = 1u; i < n; ++i) {
            seq[i] = i;
            wt[i] = waitingTime(rnd::rng);
            tQuit = std::min(tQuit, wt[i] + waitingTime(rnd::rng));
        }
        // sort and shuffle up to tQuit
        double t0 = 0.0;
        for(size_t i = 0u; i < n; ++i) {
```

```
            const double tCrit = std::min(tQuit, t0 + tAbandonPatch);

            size_t k = i;
            for(size_t j = i + 1u; j < n; ++j) if(wt[j] < wt[k]) k = j;
            if(wt[k] < tCrit) {
                std::swap(wt[i], wt[k]);
                std::swap(seq[i], seq[i + rnd::random_int(n - i)]);
                t0 = wt[i];
            }
            else {
                n = i;
                tQuit = tCrit;
                break;
            }
        }

        // interact with hosts that live in hostPatch
        size_t nEggs = 0u;
        const double maturationRate = (strategy == 3u ? generalistEfficiency : 1.0) /
         tMatureParasitoid;
        std::exponential_distribution<double> maturationTime(maturationRate);
        for(size_t i = 0u; i < n; ++i) {

            if(!(wt[i] < tQuit)) throw std::logic_error("encountering host after tQuit");

            // select focal host
            HostPatch::Host &host = hostPatch->hosts[seq[i]];

            // if the host can still be parasitised by the time it is localised...
            if(t + wt[i] < host.deadline) {
                // ...deposit an egg
                ++nEggs;
                // if the parasitisation event will affect the host...
                if(killHost) {
                    // ...sample a time for development of parasitoid offspring;
                    const double tm = t + maturationTime(rnd::rng);
                    // if offspring develops before the deadline...
                    if(tm < host.deadline)
                    {
                        //... focal parasitoid sets new host deadline
                        host.deadline = tm;
                        host.parasitoid = this;
                    }
                }
            }
        }
        // leave patch, recover and locate next patch
        if(nEggs) t += std::gamma_distribution<double>(nEggs, tRecover)(rnd::rng);
    }
    t += tQuit;
    locatePatch();
    return true;
}

void Parasitoid::getData(std::vector<size_t> &counts, std::vector<double> &sumd,
 std::vector<double> &sumdd) const
// collect data from Parasitoid objects
{
    if(strategy < 1u || strategy > 3u) throw std::logic_error("invalid value for strategy");
    size_t i = strategy - 1u;
    ++counts[i];
    sumd[i] += delta;
    sumdd[i] += delta * delta;
}

/*==============================================================================
                Implementation of the class HostPatch
==============================================================================*/
```

```
size_t HostPatch::nHostTotal = 0u;
double HostPatch::Host::lastDeadline = 0.0;

void HostPatch::collectOffspring(std::vector<Parasitoid *> &parasitoids, size_t &nHostsAlive,
 std::array<size_t, 3u> &nHostsParasitised, std::array<size_t, 3u> &nHostsKilled, const size_t
 hostType)
{
    for(Host& host : hosts) {
        if(host.parasitoid == nullptr) ++nHostsAlive;  // host survival
        else {
            const size_t strategy = host.parasitoid->strategy;
            // specialist parasitoids are incompatible with one host type
            if (hostType & strategy) {
                // successful parasitoid development
                parasitoids.push_back(new Parasitoid(host.parasitoid));
                ++nHostsParasitised[strategy - 1u];
            }
            else {
                // else: host-killing by incompatible parasitoid
                ++nHostsKilled[strategy - 1u];
            }
        }
    }
    hosts.clear();
}

void HostPatch::refill(const size_t n0)
// fills patch with n0 host individuals
{
    n = n0;
    hosts = std::vector<Host>(n);
    nHostTotal += n;
}
```

```
/*==============================================================================================
                                         FILE RANDOM.H
==============================================================================================*/

#ifndef __random__
#define __random__

#include <random>

namespace rnd
{
    extern std::mt19937_64 rng;
    unsigned int set_seed();
    unsigned int set_seed(const unsigned int);
    int random_int(const int);
    size_t random_int(const size_t);
    bool bernoulli(const double = 0.5);
    int binomial(const int, const double = 0.5);
    size_t binomial(const size_t, const double = 0.5);
    int poisson(const double = 1.0);
    size_t geometric(const double);
    double uniform(const double = 1.0);
    double normal(const double = 0.0, const double = 1.0);
    double exponential(const double = 1.0);
    int sample(const double[], const int);
}

#endif //#ifndef __random__
```

```
/*==========================================================================================
                                    FILE RANDOM.CPP
==========================================================================================*/

#include "random.h"
#include <chrono>
#include <sstream>

namespace rnd
{
    std::mt19937_64 rng;

    unsigned int set_seed()
    {
        unsigned int seed = static_cast<unsigned
         int>(std::chrono::high_resolution_clock::now().time_since_epoch().count());
        return set_seed(seed);
    }

    unsigned int set_seed(const unsigned int seed)
    {
        rng.seed(seed);
        return seed;
    }

    bool bernoulli(const double p)
    {
        return std::bernoulli_distribution(p)(rng);
    }

    int binomial(const int n, const double p)
    {
        return std::binomial_distribution<int>(n, p)(rng);
    }

    size_t binomial(const size_t n, const double p)
    {
        return std::binomial_distribution<size_t>(n, p)(rng);
    }

    int poisson(const double lambda)
    {
        return std::poisson_distribution<int>(lambda)(rng);
    }

    size_t geometric(const double p)
    {
        return std::geometric_distribution<size_t>(p)(rng);
    }

    int random_int(const int n)
    {
        return std::uniform_int_distribution<int>(0, n - 1)(rng);
    }

    size_t random_int(const size_t n)
    {
        return std::uniform_int_distribution<size_t>(0u, n - 1u)(rng);
    }

    double uniform(const double max)
    {
        return std::uniform_real_distribution<double>(0.0, max)(rng);
    }
```

```
    double normal(const double mean, const double stdev)
    {
        return stdev == 0.0 ? 0.0 : std::normal_distribution<double>(mean, stdev)(rng);
    }

    double exponential(const double lambda)
    {
        return std::exponential_distribution<double>(lambda)(rng);
    }

    int sample(const double pdf[], const int J)
    {
        return std::discrete_distribution<int>(pdf, pdf + J)(rng);
    }
}



/*==============================================================================================
                        EXAMPLE PARAMETERFILE parameters.txt
==============================================================================================*/

rng_seed_clock
r_host1                             5.0
alpha_host1                         0.001
r_host2                             5.0
alpha_host2                         0.001
t_mature_host                       10.0
t_locate_patch                      0.5
t_abandon_patch                     0.1
t_locate_host                       0.05
t_recover                           1.0
t_mature_parasitoid                 20.0
generalist_efficiency               0.5
nonreproductive_host_killing        1
mutation_rate                       1.0e-3
mutation_step                       1.0e-2
initial_parasitoid_population_size  50
number_of_iterations                1000
store_data_interval                 10
```
